# Crimean-Congo Hemorrhagic Fever, Mauritania

**DOI:** 10.3201/eid2604.191292

**Published:** 2020-04

**Authors:** Boushab Mohamed Boushab, Mamadou Kelly, Hasmiou Kébé, Mohamed Abdallahi Bollahi, Leonardo K. Basco

**Affiliations:** Kiffa Regional Hospital, Assaba, Mauritania (B.M. Boushab);; Institut National de Recherche en Santé Publique, Nouakchott, Mauritania (M. Kelly, M.A. Bollahi);; National Hospital Center of Nouakchott, Mauritania (H. Kébé);; Aix-Marseille University and IHU-Méditerranée Infection, Marseille, France (L.K. Basco)

**Keywords:** Crimean-Congo hemorrhagic fever, tickborne virus, hemorrhagic fever, viruses, arboviruses, zoonoses, Mauritania, West Africa, *Suggested citation for this article*: Boushab BM, Kelly M, Kébé H, Bollahi MA, Basco LK. Crimean-Congo hemorrhagic fever, Mauritania. Emerg Infect Dis. 2020 Apr [*date cited*]. https://doi.org/10.3201/eid2604.191292

## Abstract

The distribution of Crimean-Congo hemorrhagic fever (CCHF), a tickborne arboviral disease, is not well known in West Africa. We report 2 recent human cases of CCHF with infectious syndrome and severe bleeding in Mauritania. CCHF was diagnosed by ELISA and real time reverse transcription PCR. No secondary CCHF cases were found.

Crimean-Congo hemorrhagic fever (CCHF) occurs in Europe, Africa, the Middle East, and Asia (*1*). The virus is transmitted to humans through tick bites or direct contact with blood, secretions, or infected tissue of a viremic animal or person. The incubation period in humans is usually ≈5–6 days and hemorrhaging often occurs on the fourth or fifth day after onset of illness; ≈30% of human case-patients die. In Mauritania, CCHF was first documented in 1983 (*2*). Although several cases have been reported since, its current distribution is not well known. We report 2 cases of CCHF in 2019 in southern Mauritania.

The first patient, a 51-year-old man, a cattle breeder who resided in Tintane, Hodh Elgharbi, was admitted to Kiffa Regional Hospital, Assaba, Mauritania, on June 17, 2019, for hemorrhagic syndrome. The patient began having symptoms, including abdominal pain, bloody diarrhea, and vomiting, 5 days prior. At admission, the patient was in a coma (Glasgow coma scale 8) and had a fever (temperature 41°C), epistaxis, gingivorrhagia, diffuse ecchymosis (Figure), pallor, rapid respiratory rate (20 breaths/min), and hypotension (60/40 mm Hg). Laboratory examinations showed severe anemia (3.5 mmol/L); leucocytosis (1.3 × 10^9^ cells/L); severe thrombocytopenia (20 × 10^9^/L); prolonged prothrombin time (61%); and elevated urea (35 mmol/L), creatinine (2,298 µmol/L), alanine aminotransaminase (1.2 µkat/L), and aspartate aminotransferase (1.8 µkat/L). Rapid diagnostic tests for malaria, hepatitis B antigen, and HIV were negative. An in-house ELISA developed by Institut Pasteur de Dakar (Dakar, Senegal) was positive for CCHF virus–specific IgM and negative for yellow fever, Rift Valley fever, West Nile virus, dengue, and chikungunya (*3*). Real time reverse transcription PCR (Liferiver Bio-Tech Corp., http://www.liferiverbiotech.com) further confirmed the diagnosis. The patient was treated with ribavirin for 10 days and received several blood transfusions. He recovered and was discharged without any sequelae after 14 days. During a follow-up 2 weeks later, he was well and remained asymptomatic.

**Figure:**
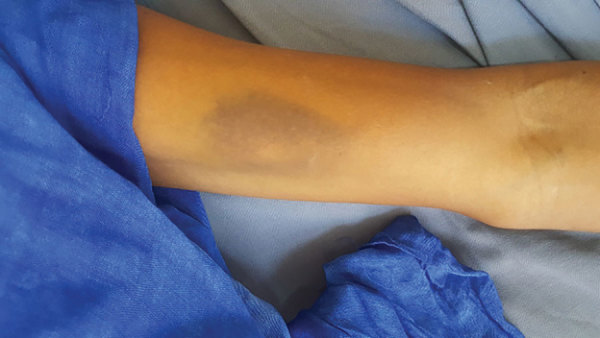
Ecchymosis on the forearm of a man diagnosed with Crimean-Congo hemorrhagic fever in Mauritania, 2019.

The second patient, a 54-year-old man, also a cattle breeder, from Guerou, Assaba, was hospitalized in a private clinic with a presumptive diagnosis of malaria and treated with quinine despite a negative blood smear. On July 9, 2019, because of altered consciousness and diffuse hemorrhagic syndrome, he was transferred to Kiffa Regional Hospital. At admission, the patient was in a coma (Glasgow coma score 7) and had epistaxis, gingivorrhagia, hematemesis, diffuse ecchymosis, gross hematuria, fever (temperature 40.5°C), and hypotension (60/40 mm Hg). Laboratory examinations showed anemia (4.5 mmol/L); low leukocyte count (1.8 × 10^9^ cells/L); severe thrombocytopenia (19 × 10^9^/L); prolonged prothrombin time (51%); and elevated renal (urea 38.2 mmol/L, creatinine 3,270 µmol/L) and liver (alanine aminotransaminase 1.37 µkat/L, aspartate aminotransferase, 1.95 µkat/L) function tests. ELISA was positive for CCHF virus (IgM positive, IgG negative) and negative for other hemorrhagic fever viruses. Real time reverse transcription PCR also was positive for CCHF virus. As in the first case, the patient was isolated from other patients and treated with ribavirin, antipyretics, blood transfusions, rehydration, and tepid sponge baths. He recovered favorably after 10 days of hospitalization. He was discharged and seen in the outpatient clinic 15 days later without any sequelae.

We did not observe ticks or tick bites during clinical examination of the patients. The most probable source of infection was close contact with infected animals. We identified all family members and hospital staff (n = 62) who came in direct or indirect contact with the 2 patients and followed them for possible secondary transmission for 3 weeks but did not observe any additional cases.

Most known human cases of CCHF in West Africa have been reported from southern Mauritania. The first documented case in West Africa occurred in 1983 in a camel and cattle breeder in Selibaby, Guidimakha region, southern Mauritania, probably after close contacts with infected camels or cattle (*2*,*4*). Subsequent reports on 6 CCHF virus–infected patients came from Rosso in the Trarza region and suggested that sheep could be a major source of transmission to humans (*5*). These sporadic cases were followed by an outbreak involving 35 persons in Nouakchott, the capital of Mauritania, including secondary infections among hospital staff, and 3 isolated cases in Brakna and Hodh Elgharbi regions in 2003 (*3*). A decade later, an unusual case of human CCHF with subdural hematoma was reported from Nouakchott (*6*). In 2015, an analysis of blood samples from cases of hemorrhagic fever during a Rift Valley fever outbreak in Mauritania showed that 6/184 (3.3%) samples, mostly from the southern part of the country, were positive for CCHF viral RNA (*7*). Most CCHF reported in humans in the region occurred during the long dry season, December–July. Animal studies also have demonstrated a high seroprevalence in cattle in Mauritania (*8*). These reports from Mauritania, together with recent findings in the neighboring countries (*9*,*10*), strongly suggest that CCHF is both enzootic and endemic in West Africa and highlight the need for better diagnostic capacity, increased awareness and knowledge of CCHF epidemiology among health providers, and regional surveillance.
